# Identification and Quantification of Bovine Digital Dermatitis-Associated Microbiota across Lesion Stages in Feedlot Beef Cattle

**DOI:** 10.1128/mSystems.00708-21

**Published:** 2021-07-27

**Authors:** Ben Caddey, Karin Orsel, Sohail Naushad, Hooman Derakhshani, Jeroen De Buck

**Affiliations:** a Department of Production Animal Health, Faculty of Veterinary Medicine, University of Calgary, Calgary, Alberta, Canada; b Ottawa Laboratory Fallowfield, Canadian Food Inspection Agency, Ottawa, Ontario, Canada; c Department of Medicine, McMaster University, Hamilton, Ontario, Canada; Colorado State University

**Keywords:** digital dermatitis, bovine, beef cattle, feedlot, *Treponema*, hoof, skin microbiota, microbiome, 16S metagenomics

## Abstract

Bovine digital dermatitis (DD) is a skin disorder that is a significant cause of infectious lameness in cattle around the world. However, very little is known about the etiopathogenesis of the disease and the microbiota associated with DD in beef cattle. In this study, we provide a comprehensive characterization of DD and healthy skin microbiota of feedlot beef cattle. We also developed and validated a novel multiplex quantitative PCR (qPCR) assay to quantify the distribution of DD-associated bacterial species across DD lesion stages. We determined the DD-associated microbiota with deep amplicon sequencing of the V3-V4 hypervariable region of the 16S rRNA gene, followed by the application of novel and existing qPCR assays to quantify species distributions of Treponema, *Porphyromonas*, *Fusobacterium*, and *Bacteroides* across lesion stages. Deep amplicon sequencing revealed that Treponema, *Mycoplasma*, *Porphyromonas*, and *Fusobacterium* were associated with DD lesions. Culturing of DD biopsy specimens identified Porphyromonas levii, Bacteroides pyogenes, and two *Fusobacterium* spp. within DD lesions. Using species-specific qPCR on DD lesion DNA, we identified P. levii in 100% of active lesion stages. Early-stage lesions were particularly associated with Treponema medium, T. phagedenis, and *P. levii*. This study suggests a core DD microbial group consisting of species of Treponema, *Fusobacterium*, *Porphyromonas*, and *Bacteroides*, which may be closely tied with the etiopathogenesis of DD. Further characterizations of these species and *Mycoplasma* spp. are necessary to understand the microbial factors involved in DD pathogenesis, which will help elucidate DD etiology and facilitate more targeted and effective mitigation and treatment strategies.

**IMPORTANCE** Previous work, primarily in dairy cattle, has identified various taxa associated with digital dermatitis (DD) lesions. However, there is a significant gap in our knowledge of DD microbiology in beef cattle. In addition, characterization of bacteria at the species level in DD lesions is limited. In this study, we provide a framework for the accurate and reproducible quantification of major DD-associated bacterial species from DNA samples. Our findings support DD as a polymicrobial infection, and we identified a variety of bacterial species spanning multiple genera that are consistently associated with DD lesions. The DD-associated microbiota identified in this study may be capable of inducing the formation and progression of DD lesions and thus should be primary targets in future DD pathogenesis studies.

## INTRODUCTION

Bovine digital dermatitis (DD) is an infectious skin lesion affecting cattle around the world and was first described in the 1970s (1, 2). DD lesions, primarily localized to the skin between the heel bulbs on the hind legs, are a significant contributor to infectious lameness resulting in major production and economic losses in dairy cattle (3–5). Since its original description, DD has been primarily studied as a dairy industry issue, with cow-level prevalence estimates ranging from 3% to 23% (6, 7); however, DD has recently been emerging in feedlot beef cattle populations (2), as reported by Kulow et al., where that approximately 50% of feedlot cattle on one farm experienced a DD lesion during their study period (8). Management practices mainly focus on broad-spectrum antimicrobials, most commonly topical applications of oxytetracycline or copper sulfate; however, treatment of DD has unsatisfactory cure rates of as low as 9% (9), beckoning calls for a greater understanding of DD etiopathogenesis and more targeted treatments.

The etiological agents of DD are not yet fully identified, as lesions are polymicrobial in nature and contain various fastidious anaerobic bacteria, explaining the lack of dedicated culture methods and the insufficient characterization of species and type strains necessary to understand bacterial pathogenesis mechanisms. Spirochetes are the most consistent bacterial group associated with DD lesions (10–12). Spirochetes of various Treponema species and phylotypes are identified in DD lesions, and individual lesions normally contain different combinations of Treponema spp. at different proportions (13, 14). In addition to Treponema spp., multiple studies of dairy cattle DD lesions identify *Porphyromonas* (15), *Dichelobacter* (16), *Guggenheimella* (17), *Bacteroides* (18), *Fusobacterium* (19), *Mycoplasma* (20), and many other genera as being associated with DD lesions, further supporting a polymicrobial causation of DD.

There is limited knowledge on the presence and the population dynamics of bacterial species throughout DD stages. Most recent DD microbiology studies employ high-throughput sequencing strategies and have expanded our understanding of DD, but they fail to reliably classify or quantify species-level taxa (21). Recently, Beninger et al. quantified four major Treponema spp. in DD lesions of dairy cattle by species-specific multiplex quantitative PCR (qPCR) and identified Treponema phagedenis, T. pedis, and T. medium as highly correlated with DD disease development and progression (22). There remains a significant number of additional DD-associated Treponema spp. that require further characterization to better understand their potential involvement in disease pathogenesis. In addition, no validation of the population dynamics of non-Treponema species exists in the DD literature, and thus, speculation of their role in DD etiopathogenesis is limited.

In contrast to dairy breeds, the microbiology of DD lesions in beef breeds is largely unexplored. DD lesions in beef cattle are associated with the presence of different Treponema phylotypes as well as Fusobacterium necrophorum and Dichelobacter nodosus (23, 24). However, there is no knowledge currently on the quantities and distributions of any DD-associated bacteria in beef cattle DD lesions. Due to the differences in housing and management practices between dairy and feedlot systems, there is insignificant evidence at this time to suggest that the microbial community structure in dairy cattle DD accurately represents that of beef cattle DD microbiota. Without a full comprehensive identification of the DD-associated microbiota in beef cattle, we cannot reliably extrapolate our existing knowledge of DD microbiology in dairy cattle lesions.

This study aimed to target the gaps in the knowledge of DD microbiology in feedlot beef cattle. As DD is an emerging issue in feedlot beef cattle, it was crucial to perform a comprehensive microbiological assessment of DD lesions in this cattle population. Microbiota members associated with DD lesions and healthy skin of beef cattle were identified. To better understand which bacterial species are associated with DD lesion formation and progression, bacterial population dynamics were quantified throughout DD lesion stages using existing and newly developed species-specific real-time quantitative PCR assays.

## RESULTS

Altogether, we collected and analyzed the microbial compositions of 120 skin biopsy specimens. We collected 40 biopsy specimens from healthy skin (M0), 8 biopsy specimens from M1 lesions, 38 biopsy specimens from ulcerated M2 lesions, 4 biopsy specimens from healing M3 lesions, 20 biopsy specimens from M4 lesions, and 10 biopsy specimens from active M4.1 lesions.

### Deep amplicon sequencing of the V3-V4 hypervariable region.

**FIG 1:**
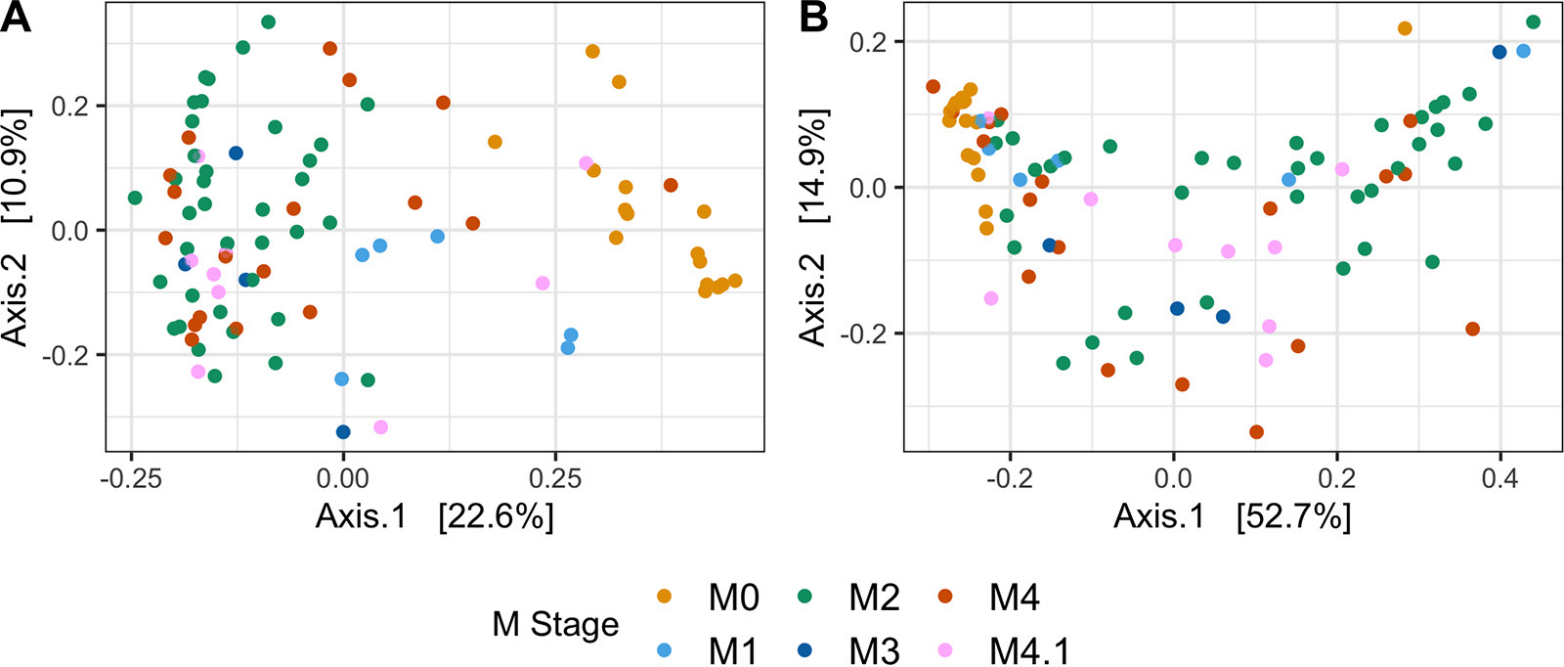
Principal-coordinate analyses of unweighted (A) and weighted (B) UniFrac distances. Samples are colored based on the M stage of DD lesions.

Out of 120 skin biopsy specimens obtained in this study, 98 resulted in successful amplification, based on gel electrophoresis of the nested V3-V4 hypervariable region PCR assay, and were then sequenced on the Illumina MiSeq platform (M0, *n* = 20; M1, *n* = 8; M2, *n* = 37; M3, *n* = 4; M4, *n* = 19; M4.1, *n* = 10). A total of 4,860,255 sequences passed initial quality filtering, and after inferring amplicon sequence variants (ASVs) and removing chimeras, 2,994,368 sequences were used for taxonomic classification. No sequences from DNA extraction controls remained after quality filtering and DADA2 processing (see Fig. S1A in the supplemental material). Negative controls (DNase/RNase-free water) contained relatively fewer reads, on average, than healthy skin and DD samples and represented only two ASVs from *Proteobacteria* (Fig. S1A and B). After removing sequences with low numbers of reads (<500), 90 samples remained for microbiota analysis (M0, *n* = 16; M1, *n* = 6; M2, *n* = 37; M3, *n* = 4; M4, *n* = 18; M4.1, *n* = 9). Permutational multivariate analysis of variance (PERMANOVA) on weighted and unweighted UniFrac distances each identified a significant difference (*P* = 0.001) in microbial compositions among M stages (Fig. 1A and B).

10.1128/mSystems.00708-21.1 FIG S1 Analysis of Illumina MiSeq read counts in sequencing controls. (A) Sequences for each group were counted after raw reads were processed with DADA2 and are displayed as mean read counts ± SD. Labels represent, in order, healthy skin (M0), DD lesion samples (M1, M2, M3, M4, and M4.1), a negative control (NC) consisting of RNase/DNase-free water, and a DNA extraction control (EC). (B) Microbial composition of negative controls based on phylum-level taxonomy. Download FIG S1, TIF file, 8.4 MB. Copyright © 2021 Caddey et al. 2021 Caddey et al. https://creativecommons.org/licenses/by/4.0/ This content is distributed under the terms of the Creative Commons Attribution 4.0 International license.

**FIG 2:**
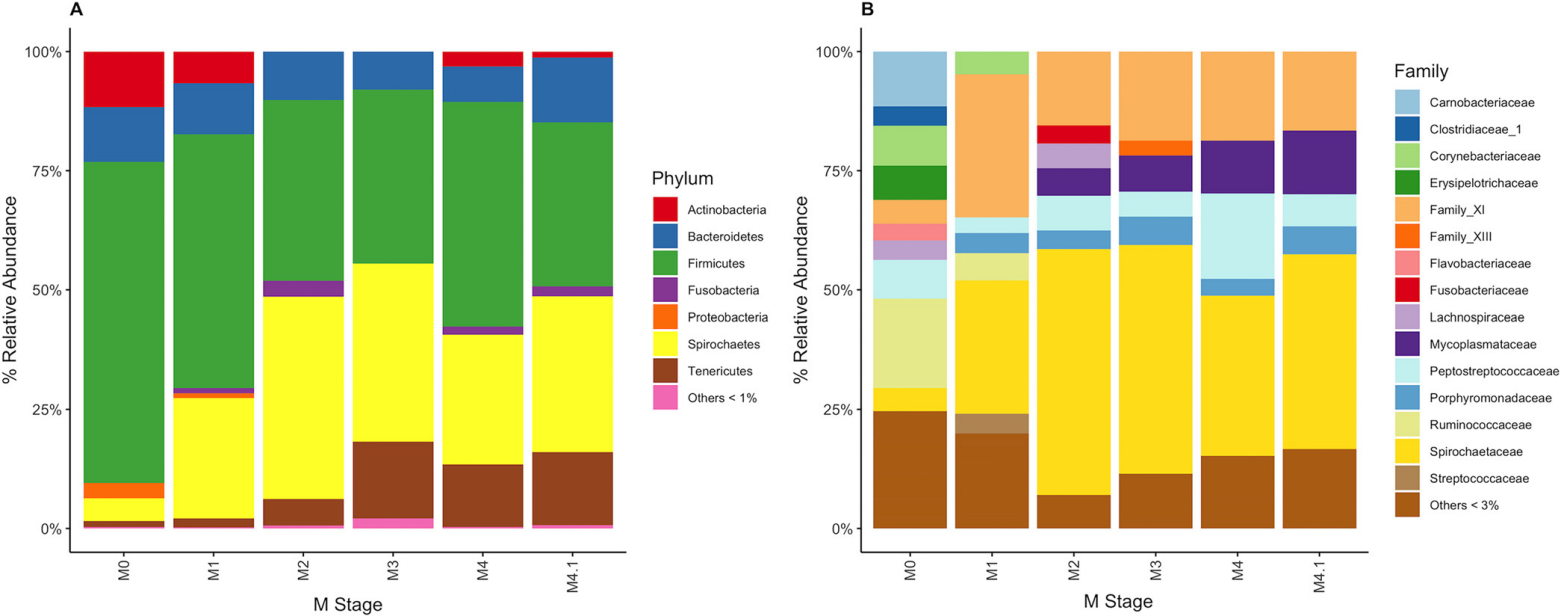
Percent relative abundance of bacteria within each M stage. (A) Bacteria were grouped based on taxonomy at the phylum level, and phyla with a <1% relative abundance were grouped. (B) Bacteria were grouped based on taxonomy at the family level, and families with a <3% relative abundance were grouped.

Healthy skin primarily contained *Actinobacteria*, *Bacteroidetes*, *Proteobacteria*, and, predominantly, *Firmicutes* (Fig. 2A). Compared to healthy skin, DD lesions across all M stages had a high relative abundance of *Spirochaetes*, accompanied by a high relative abundance of *Tenericutes* (Fig. 2A). *Fusobacteria* were also associated with DD lesions, were relatively absent in healthy skin, and peaked at an ∼3.4% relative abundance in M2 lesions (Fig. 2A). For family-level taxonomic grouping (Fig. 2B), M0 skin contained a higher number of unique bacterial families than all other M stages. Of the taxonomically classifiable sequences, *Spirochaetaceae* were the most abundant in DD lesions (Fig. 2B). There were also higher relative abundances of *Porphyromonadaceae*, *Mycoplasmataceae*, family XI, and *Fusobacteriaceae* in DD lesions than in healthy skin (Fig. 2B). No obvious visual differences were apparent in relative abundances between M stages for these DD-associated bacteria. *Mycoplasmataceae*, however, had their highest relative abundance in M3, M4, and M4.1 lesions (Fig. 2B). *Fusobacteriaceae* had a higher relative abundance in M2 lesions than in the other disease stages, having a relative abundance of only >3% in M2 lesions (Fig. 2B).

**FIG 3:**
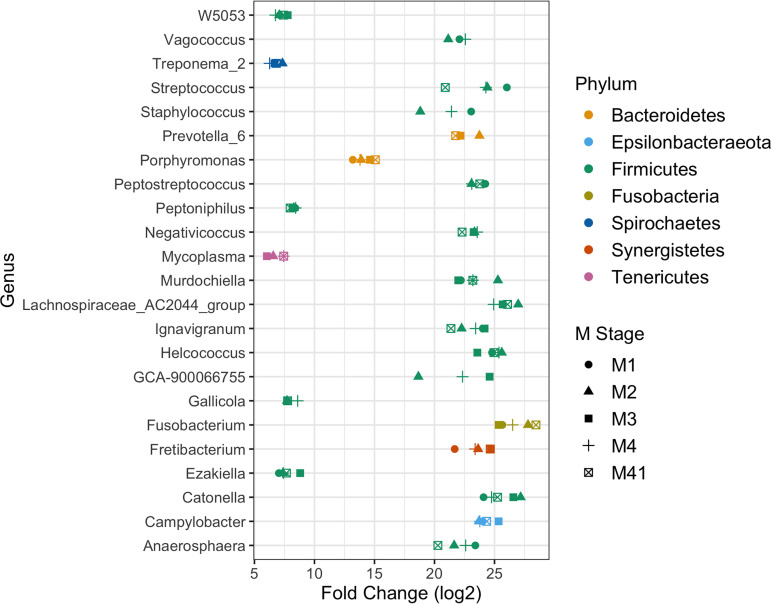
Differential abundance analysis of genera associated with DD lesions. DESeq2-normalized sample counts were used to compare genus fold changes in abundance for DD M stages (M1, M2, M3, M4, and M4.1) against stage M0 (healthy) samples. Only genera that were statistically significant (*P* < 0.01) in at least three DD M stages compared to healthy skin are shown.

Using DESeq2, differential abundance analysis of DD lesions compared to healthy skin identified a group of genera strongly associated with DD lesions (Fig. 3). Treponema, *Porphyromonas*, *Mycoplasma*, and *Fusobacterium* relative abundances were significantly higher (*P* < 0.01) in most of the DD M stages than in healthy skin (Fig. 3). In addition, many genera that are members of the *Firmicutes* phylum had significantly higher (*P < *0.01) counts in most DD lesions than in healthy skin (Fig. 3).

**FIG 4:**
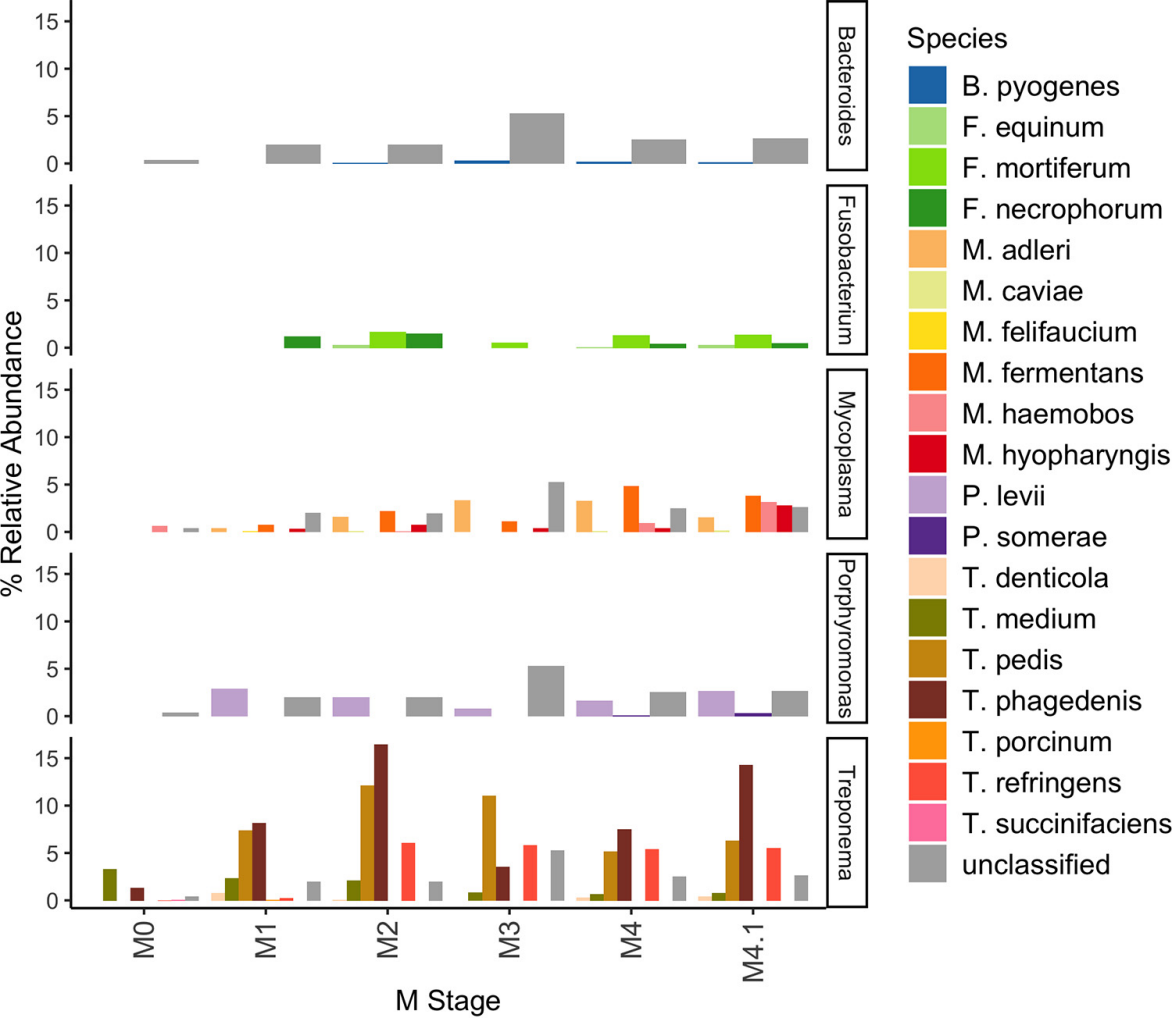
Percent relative abundance of bacterial species within each M stage. ASVs that could not be classified at the species level were grouped as unclassified within each genus. Bacterial species outside the five genera of interest (*Bacteroides*, *Fusobacterium*, *Mycoplasma*, *Porphyromonas*, and Treponema) are not shown.

Species-level classification was pursued for members of the genera *Bacteroides*, *Fusobacterium*, *Mycoplasma*, *Porphyromonas*, and Treponema. Interestingly, *Fusobacterium* was the only genus to not have unclassified reads, while all other genera had large proportions of unclassified ASVs. For *Bacteroides*, only B. pyogenes was identified in M2 and M4 lesions at a comparatively low relative abundance (Fig. 4). Fusobacterium mortiferum was not detectable in M1 lesions but was relatively more abundant than Fusobacterium necrophorum in all other DD stages (Fig. 4). Of all *Mycoplasma* spp. identified, M. fermentans was the primary *Mycoplasma* sp. within M2 lesions, whereas in other M stages (M4 and M4.1), multiple species of *Mycoplasma* became relatively abundant (Fig. 4). The distribution of Treponema spp. appeared highly dynamic across M stages, beginning with *T. medium* as the predominant species present in healthy skin, and *T. pedis*, *T. phagedenis*, and T. refringens were relatively abundant in all DD lesion stages (Fig. 4).

### Identification of bacterial isolates from skin biopsy specimens.

Bacterial culture was attempted for all skin biopsy specimens obtained. A total of 198 isolates from 69 biopsy specimens were successfully cultured and identified. Bacteria that were cultured from DD lesions and were not isolated from healthy skin biopsy specimens are shown in Table S2. A number of species from the genera *Fusobacterium* and *Porphyromonas* were isolated from all M stages of DD lesions but were not cultured from M0 biopsy specimens. In addition, *B. pyogenes* was identified in all M stages of DD lesions but was not isolated from M0 samples.

10.1128/mSystems.00708-21.4 TABLE S2 Number of species isolates collected from each DD lesion stage. Download Table S2, DOCX file, 0.01 MB. Copyright © 2021 Caddey et al. 2021 Caddey et al. https://creativecommons.org/licenses/by/4.0/ This content is distributed under the terms of the Creative Commons Attribution 4.0 International license.

### Quantitative real-time PCR design.

Isolates of *B. pyogenes*, *Fusobacterium* sp., and Porphyromonas levii were collected and identified by full-length 16S rRNA sequence alignment (Table S2). The best match of the *Fusobacterium* sp. 16S rRNA gene sequence was against F. mortiferum (96.97% identity). These three species isolated from biopsy specimen cultures matched the DD-associated microbiota identified by deep amplicon sequencing (Fig. 4) and thus were targeted for qPCR development. To demonstrate the accuracy of the qPCR, we spiked 5 × 10^4^ copies of target DNA directly into purified biopsy specimen DNA, in which the maximum difference observed between the actual DNA copies present and the mean qPCR output was 7 × 10^3^, or 14% (Table S3).

10.1128/mSystems.00708-21.5 TABLE S3 DNA spike-in to evaluate the performance of multiplex qPCR detection directly from biopsy specimen DNA. Download Table S3, DOCX file, 0.01 MB. Copyright © 2021 Caddey et al. 2021 Caddey et al. https://creativecommons.org/licenses/by/4.0/ This content is distributed under the terms of the Creative Commons Attribution 4.0 International license.

### Species absolute quantification directly from purified biopsy specimen DNA.

**FIG 5:**
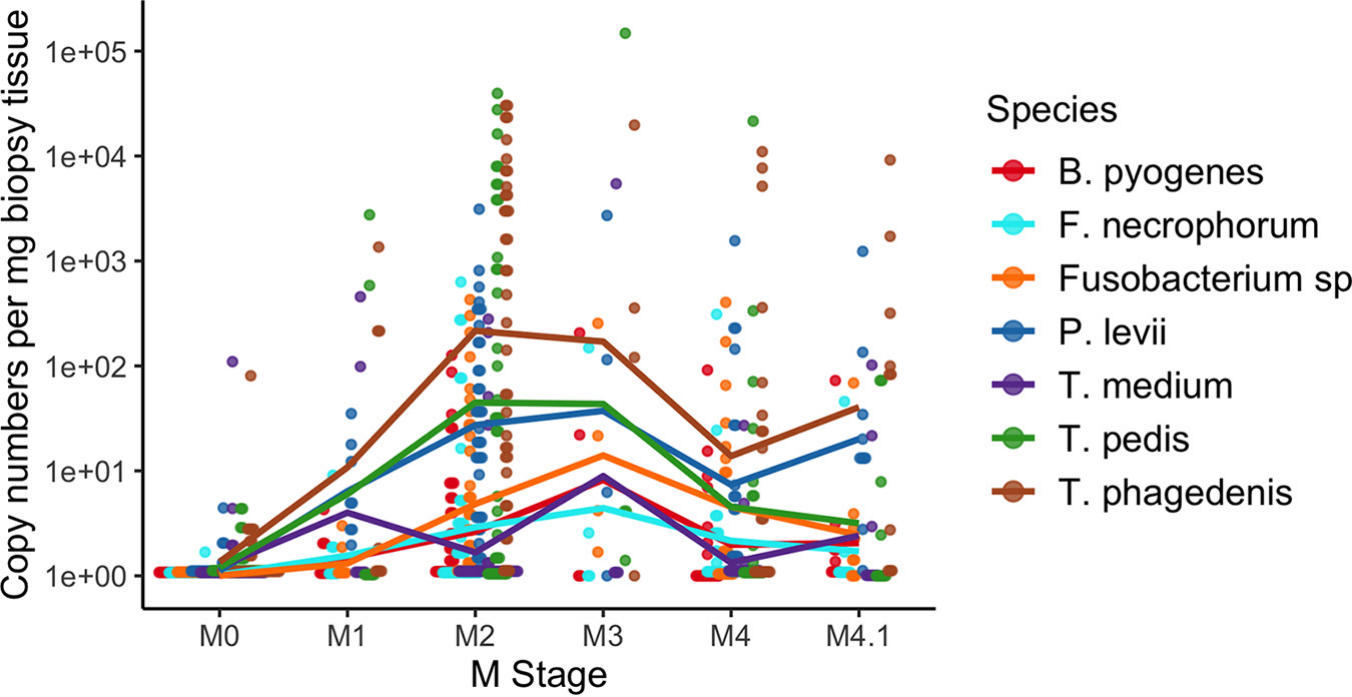
Absolute abundance of each species across all M stages. Species copy numbers were standardized by the weight of the biopsy tissue. Each dot and color represent a different sample and a bacterial species, respectively. Lines are present for easy visual tracking of mean species copy numbers but do not represent a linear or continuous relationship between M stages.

A total of 120 purified biopsy DNA samples (M0, *n* = 40; M1, *n* = 8; M2, *n* = 38; M3, *n* = 4; M4, *n* = 20; M4.1, *n* = 10) were used for the absolute quantification of *T. phagedenis*, *T. pedis*, *T. medium*, T. denticola, *Fusobacterium* sp., F. necrophorum, *B. pyogenes*, and P. levii. The absolute abundance was quantified for a total of 8 DD-associated species. Treponema denticola was detected in only 5 samples due to the qPCR requiring at least 10^3^ gene copies for detection. Thus, T. denticola was not included in further analyses. All DNA extraction controls had nondetectable levels of each species. Most of the species quantified were significantly more abundant (*P < *0.05) in DD lesions than in healthy skin, except for *T. medium*, which had no significant differences in abundance across any M stage (Table 1). The majority of species had their highest abundances in M2 and M3 lesions (Table 1 and Fig. 5).

**TABLE 1:** Bacterial genomic copies per milligram of biopsy tissue of each bacterial species at each M stage

Lesion stage	Mean no. of genomic copies/mg biopsy tissue ± SD^*a*^						
	*T. phagedenis*	*T. pedis*	*T. medium*	F. necrophorum	*Fusobacterium* sp.	*P. levii*	*B. pyogenes*
M0	2.28 ± 12.5 A	0.333 ± 0.829 A	2.87 ± 17.2 A	0.023 ± 0.110 A	0.004 ± 0.029 A	0.181 ± 0.579 A	0.005 ± 0.026 A
M1	223 ± 468 B	417 ± 965 AB	69.1 ± 160 A	1.24 ± 2.79 B	0.426 ± 0.708 B	9.32 ± 11.4 BC	0.700 ± 1.14 B
M2	4.56 × 10^3^ ± 8.42 × 10^3^ C	3.19 × 10^3^ ± 8.08 × 10^3^ B	14.8 ± 55.8 A	36.0 ± 118 B	34.6 ± 89.2 BC	186 ± 520 B	8.88 ± 24.8 B
M3	5.05 × 10^3^ ± 9.78 × 10^3^ ABC	3.70 × 10^4^ ± 7.39 × 10^4^ B	1.36 × 10^3^ ± 2.72 × 10^3^ A	37.3 ± 73.6 B	69.5 ± 123 C	708 ± 1.34 × 10^3^ BC	56.6 ± 99.6 B
M4	1.22 × 10^3^ ± 3.05 × 10^3^ B	1.10 × 10^3^ ± 4.80 × 10^3^ B	1.55 ± 5.84 A	17.2 ± 69.0 B	35.6 ± 94.8 BC	112 ± 348 C	6.11 ± 20.3 B
M4.1	1.15 × 10^3^ ± 2.87 × 10^3^ BC	15.2 ± 29.9 AB	12.4 ± 31.8 A	4.70 ± 14.1 B	7.59 ± 21.2 BC	147 ± 384 BC	7.60 ± 22.5 B

a Different letters within a column indicate a significant difference (*P* < 0.05).

All species tested by qPCR were detectable in the majority of DD lesions, except for *T. medium*, which was detectable in only approximately 40% of DD lesions but was present in 62% of M1 lesions (Table 2). In active DD lesions, *P. levii* was detectable by qPCR in all samples and detectable in 38% of healthy skin samples (Table 2). Similarly, *T. phagedenis* was detectable in the majority of samples from all M stages, including healthy skin (Table 2). Of all the species that were detectable in the majority of DD lesion samples, *Fusobacterium* sp. was detected in the smallest amount of healthy skin samples, at 2.5% (Table 2).

**TABLE 2:** Percentages of samples with detectable^*a*^ amounts of each bacterial species

Lesion stage (no. of samples)	% of samples with detectable species present						
	*T. phagedenis*	*T. pedis*	*T. medium*	F. necrophorum	*Fusobacterium* sp.	*P. levii*	*B. pyogenes*
M0 (40)	68	32	25	10	2	38	5
M1 (8)	100	38	62	50	50	100	62
M2 (38)	97	76	29	50	76	100	60
M3 (4)	75	100	75	50	100	75	50
M4 (20)	75	65	40	55	60	85	35
M4.1 (10)	80	40	40	50	70	100	70

Total DD (80)	89	66	39	51	70	95	55

a Detectable by qPCR.

**FIG 6:**
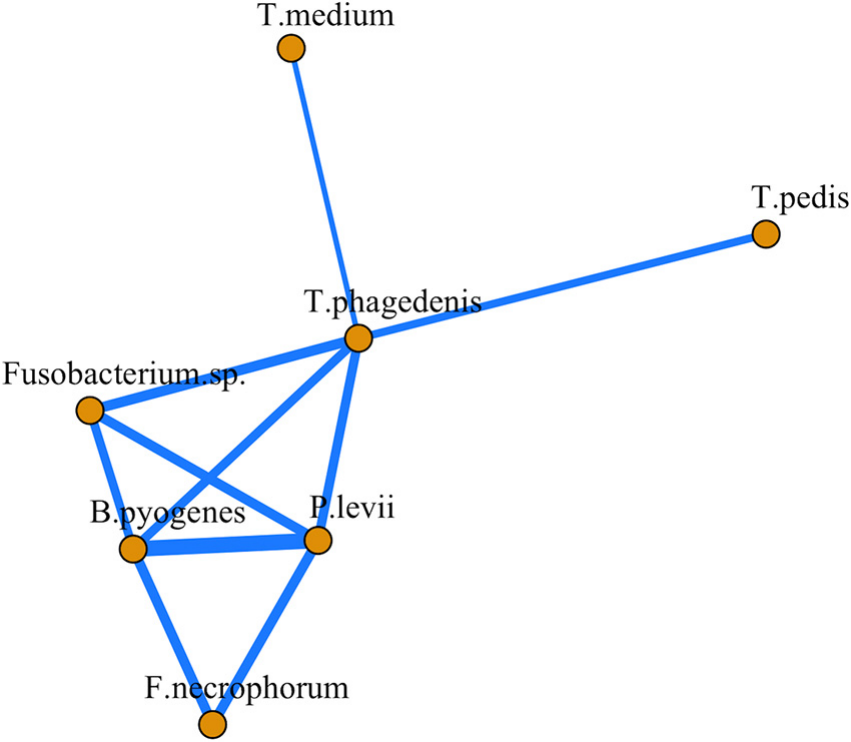
Correlation network analysis of species absolute abundances in DD lesions. Significant pairwise Spearman correlations (*P* < 0.05) were included in the network analysis. The thickness of each line is proportional to the level of correlation. All correlations included were positive.

Spearman rank correlations performed on absolute abundances in DD lesions identified *B. pyogenes* and *P. levii* as having the strongest associations among all pairwise species combinations (Fig. 6; Fig. S2). Negative correlations were identified only in *T. medium* pairwise comparisons with both *Fusobacterium* species (Fig. S2). In addition, *T. medium* had significant pairwise correlations (*P < *0.05) with only *T. phagedenis* (Fig. 6). Treponema phagedenis was the only Treponema species to have significant correlations (*P < *0.05) with non-Treponema species (Fig. 6). *Fusobacterium* sp. and F. necrophorum abundances were not significantly correlated (Fig. 6).

10.1128/mSystems.00708-21.2 FIG S2 Pairwise associations between log copy numbers of species in DD lesions. Species copy numbers per milligram of biopsy tissue were transformed with the natural logarithm after the addition of a pseudocount of 1. Each dot represents the bacterial copy number in separate samples, split into active (M1, M2, and M4.1) and inactive (M3 and M4) DD lesions. Spearman rank correlation coefficients measured the degree of association between each species pair in all DD lesions. Download FIG S2, TIF file, 11.5 MB. Copyright © 2021 Caddey et al. 2021 Caddey et al. https://creativecommons.org/licenses/by/4.0/ This content is distributed under the terms of the Creative Commons Attribution 4.0 International license.

### Absolute abundances of species in healthy skin across farms.

Healthy skin samples from three separate feedlots were acquired: two feedlots had active cases of DD at the time of sampling, and one feedlot had no active cases of DD during the sampling period. In the feedlot with no active DD cases, *T. phagedenis*, *T. medium*, F. necrophorum, *B. pyogenes*, and *P. levii* had significantly lower abundances (*P < *0.05) than in healthy skin samples from both active DD feedlots (Table S4). In addition, *T. medium*, F. necrophorum, and *B. pyogenes* were detectable in zero samples from the feedlot without active DD cases (Table S5).

10.1128/mSystems.00708-21.6 TABLE S4 Bacterial genomic copies (means ± SD) per milligram of biopsy tissue of each bacterial species at each farm for healthy (M0) samples. Download Table S4, DOCX file, 0.01 MB. Copyright © 2021 Caddey et al. 2021 Caddey et al. https://creativecommons.org/licenses/by/4.0/ This content is distributed under the terms of the Creative Commons Attribution 4.0 International license.

10.1128/mSystems.00708-21.7 TABLE S5 Percentage of healthy skin biopsy specimens with detectable amounts of target bacterial species at each farm. Download Table S5, DOCX file, 0.01 MB. Copyright © 2021 Caddey et al. 2021 Caddey et al. https://creativecommons.org/licenses/by/4.0/ This content is distributed under the terms of the Creative Commons Attribution 4.0 International license.

### Comparison of qPCR and deep amplicon sequencing quantification methods.

**FIG 7:**
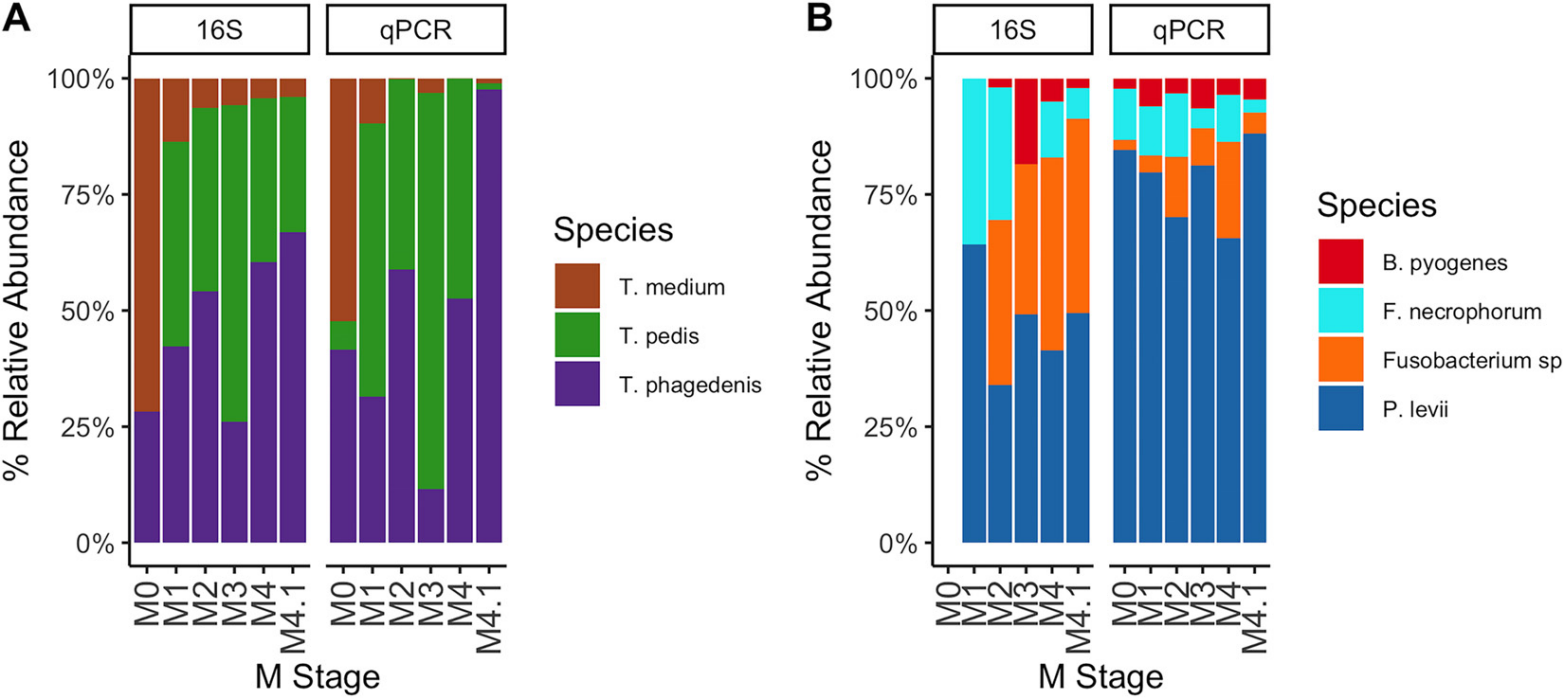
Percent relative abundances of species in each M stage compared by quantification method. Species were separated according to Treponema species (A) and non-Treponema species (B). Relative abundances were compared between deep amplicon sequencing of the V3-V4 hypervariable region of the 16S rRNA gene and species-specific qPCR quantification. *Fusobacterium* sp. targeted by qPCR and *F. mortiferum* identified by deep amplicon sequencing were both labeled as *Fusobacterium* sp.

On average, Treponema made up the majority of the detectable DD lesion microbiota, regardless of the quantification method (Fig. 2 and Fig. 5). Data from deep amplicon sequencing appeared to consistently overrepresent *T. medium* across all M stages compared to qPCR abundances (Fig. 7). Abundance dynamics for *T. pedis* and *T. phagedenis* were relatively comparable across M stages for both quantification methods, except for a relative overrepresentation of *T. phagedenis* in the M4.1 stage as measured by qPCR (Fig. 7). Deep amplicon sequencing also showed a higher relative abundance of *Fusobacterium* spp. than with qPCR quantification, which instead favored *P. levii* abundance over all other non-Treponema species tested (Fig. 7).

## DISCUSSION

In this study, we present a microbiota strongly associated with DD lesions of feedlot beef cattle. The majority of the DD lesion microbiota comprised Treponema spp., namely, *T. phagedenis* and *T. pedis*; meanwhile, *P. levii*, *B. pyogenes*, and the presence of two different *Fusobacterium* spp. also differentiated DD lesions from healthy skin. Most of these potential DD pathogens were significantly higher in abundance in all DD M stages than in normal healthy skin. These data provide evidence of DD lesion formation and development as a potential outcome of the prevalence and abundance dynamics of these key species across DD M stages.

Overall, the beef DD lesion microbiota did not have drastic differences compared to the dairy DD lesion microbiota identified in previous studies (15, 20). Primary DD-associated members of the microbiota in beef cattle, namely, Treponema, *Porphyromonas*, *Mycoplasma*, and *Fusobacterium*, have been identified in dairy DD studies as well (20, 25, 26). We also identified significantly higher relative abundances of some *Firmicutes* taxa in DD lesions than in healthy skin in beef breeds. In contrast, there is little evidence for the involvement of *Firmicutes* members in DD progression of dairy cattle, which have low relative abundances and overall decreasing counts as lesions progress (15, 20).

Treponema spp. are consistently argued as being one of the main causative agents of DD, and we provide further evidence of their presence and potential involvement, particularly for *T. phagedenis* and *T. pedis*, which were prevalent in beef cattle DD lesions. We were able to identify seven different Treponema spp. that were present in DD lesions from classifiable V3-V4 hypervariable region sequences, supporting previous hypotheses that multiple strains and species of Treponema can play a role in DD development. We were able to accurately quantify the absolute abundances of three of these Treponema spp. by qPCR; however, there are additional DD-associated species, such as *T. refringens* (20, 25), that are outside the range of existing qPCR assays and require further study to validate their associations with DD lesion stages.

We identified that early-stage (M1) DD lesions are associated with the presence of Treponema, namely, *T. phagedenis* and *T. medium*, relative to healthy skin, suggesting the potential importance of these Treponema spp. in early lesion development. Of note, *T. medium* was significantly associated with only M1 lesions, suggesting that *T. medium* might contribute to initiating but not sustaining lesions. In contrast, *T. phagedenis* abundance was associated with nearly all DD M stages and was the only Treponema species to have significant correlations with non-Treponema species abundances. In this analysis, *T. phagedenis* appeared to be the potential interactive link between Treponema spp. and other genera, although this could be a product of the relatively high *T. phagedenis* abundance and prevalence compared to most other species examined. Further study is warranted on the potential metabolic interactions between DD-associated species.

Our qPCR assay identified *P. levii* in all active stages of DD lesions sampled in feedlot beef cattle and has been identified in DD lesions in previous studies via high-throughput sequencing and fluorescent *in situ* hybridization (FISH) (15, 25). Porphyromonas levii has been previously dismissed as a mere secondary opportunistic invader based on its predominant superficial location within the dermis of DD lesions (15, 25), potentially reducing its overall effect on DD lesion formation. However, a study quantifying bacterial gene expression patterns in DD lesions supports the potential involvement of *P. levii* in DD development (27). Thus, it is possible that *P. levii* plays a role in influencing the overall metabolic processes of the DD microbiota, especially in active lesion stages, which certainly warrants further investigation.

The identification of F. necrophorum, which is considered the primary causative agent of bovine foot rot (28), in DD lesions is not novel; however, the finding of an additional *Fusobacterium* sp. by culturing DD biopsy specimens has not been observed previously, to the best of our knowledge. Both *Fusobacterium* spp. quantified in this study had similar absolute abundances throughout each M stage, and correlation analysis identified no significant correlation between the two species, which suggests that these species are randomly dispersed across different lesions in relation to each other. Therefore, if there is a causative role for *Fusobacterium* in DD, it is possible that there may be limited functional differences between *Fusobacterium* spp. in DD development. Investigations into homologues of the main virulence factors between the two *Fusobacterium* spp. can help identify whether or not the species have unique roles in lesions. Given that F. necrophorum has been shown to generate mixed-species biofilms with *P. levii* to impair the neutrophil response (29), it is possible that the *Fusobacterium* sp. that we isolated in this study may interact in a similar way.

Healthy skin microbiota varied significantly across different feedlots with and without active DD cases at the time of sampling. Of interest, *T. medium*, F. necrophorum, and *B. pyogenes* were detectable in healthy skin only in animals on a feedlot with active cases of DD and were undetectable in feedlots without DD at the time of sampling. This finding opens a multitude of avenues for investigating the transmission dynamics of DD as well as shedding additional light on potential pathogenesis that establishes early-stage lesions in natural DD progression. These results also illuminate the possible connection of farm- and animal-based risk factors with the microbial community dynamics from healthy to DD skin. However, with the low number of feedlots included in this study and the numerous different management practices between farms, there currently does not exist sufficient validity to make significant conclusions on these findings.

The methodology and findings presented in this study provide a crucial advancement in our understanding of bovine DD microbiology. The combined use of deep amplicon sequencing and culture methods to identify key DD-associated bacterial species and then the development of a novel multiplex qPCR to validate their population dynamics across lesion stages provide a deeper and more thorough understanding of DD than any of these techniques alone. Along with the rise in high-throughput sequencing strategies to identify DD-associated microbiota comes significant limitations and biases affecting the validity of the findings, including various copy numbers of target amplicons (i.e., 16S rRNA gene) across taxa, uneven sampling depth, primer bias, low taxonomic resolution, and variation in bioinformatic processing (30). Knowledge of the microbial community is required beforehand to develop an appropriate mock community to quantify some of these biases, but this is not always possible when studying a novel ecological system. Real-time PCR can provide a more reliable quantification of a narrow subset of species and serves as an appropriate validation of deep amplicon sequencing results (31). Our method of selecting species-specific genes for qPCR targets leads to a specific reaction targeting individual species. However, this method is validated on publicly available genomes and locally derived strains; thus, it cannot be guaranteed to efficiently amplify all global strains of each species, and therefore, this qPCR should be consistently tested as more genomes become available for each species.

The combination of qPCRs used in this study targets a group of bacteria associated with DD pathogenesis, but not all species of interest could be targeted. It is critical to involve additional Treponema spp., but perhaps more importantly, we need a better understanding of the *Mycoplasma* species dynamics in DD lesions. Using deep amplicon sequencing, we identified that the relative abundance of *Mycoplasma* is highest in M4 and M4.1 lesions; therefore, further characterization of *Mycoplasma* spp. may uncover significant links in the DD etiopathogenesis of chronic lesions. *Mycoplasma* was more recently implicated in DD pathogenesis (15, 20); however, very little validated information on the species involved exists outside M. fermentans, which was identified in the majority of DD lesions in one study by PCR (15). There is a lack of targeted culture methods to successfully isolate *Mycoplasma* from DD tissue, and thus, there are no publicly available genomes from *Mycoplasma* species isolated from DD tissue or even other related diseases of the hoof area. Genomes derived from DD-relevant isolates are essential for developing robust methods to study their population dynamics throughout lesion development.

### Conclusion.

We demonstrated a combination of bacterial species strongly associated with polymicrobial DD lesions. The abundance of Treponema, *Fusobacterium*, *Bacteroides*, and *Porphyromonas* strongly differentiated DD lesions from healthy skin. The combination of methodologies and the multiplex qPCR performed in this study targets a critical need in DD research for the identification of species involved in DD lesions. Using this approach, we provide an accurate and sensitive method for the quantification of these potential DD pathogens from DNA samples. Further investigations into additional species from other genera and especially further characterization of additional Treponema and *Mycoplasma* species can facilitate significant leaps in identifying etiopathological agents. This study along with future characterizations will be necessary to fully understand the microbiological factors involved in DD progression and lead to the development of more effective mitigation and treatment strategies.

## MATERIALS AND METHODS

### Sampling strategy and biopsy specimen collection.

Beef cattle from three feedlots in southern Alberta, Canada, were sampled for DD. Forty animals each from a total of 11 separate outdoor pens were monitored throughout the feedlot cycle from fall arrival starting November 2018 to September 2019. Each pen was examined during three separate events throughout the year, in which six study animals per pen were restrained in stand-up chutes and hind feet were lifted and inspected for DD lesions. The six animals per pen for each of the three sampling events were selected based on predicted DD status, determined by pen walks prior to the sampling days, with a priority to select animals with DD lesions and those that were not previously sampled. All lesions were classified according to the M stage scoring method developed by Döpfer et al. (10) and modified by Berry et al. (32). Briefly, there are 5 different M stages: the M1 stage is a small (<2-cm) circumscribed and ulcerative lesion, M2 lesions are large ulcerative lesions compared to M1 lesions, M3 lesions are described as a healing stage with a rubbery scab covering the lesion, M4 lesions are chronic and have definitive hyperkeratotic growth with raised papilliform projections, and M4.1 lesions are chronic with a small ulcerative focus. All skin free from visible DD lesions was classified as healthy (M0). Upon inspection of feet and classification of the lesion stage, the lesion area was washed with water to remove all manure/debris before biopsy sample collection. Because the majority of feet were classified as healthy (M0), these were systematically sampled for every 2nd and 5th animal without DD that passed through the chute, whereas DD lesions were collected from each foot as encountered. After 3 ml of lidocaine (lidocaine HCl 2%; Zoetis Canada Inc., Kirkland, Quebec, Canada) was subcutaneously injected, samples were collected with a 4-mm biopsy punch (Integra Miltex; Integra Life Sciences Corporation, York, PA, USA). All biopsy cores were immediately placed upright in anaerobic transport medium (ATM; Anaerobe Systems, Morgan Hill, CA, USA) and transported to be processed at the laboratory within 8 h of sampling. All animal use was approved by the University of Calgary Veterinary Sciences Animal Care Committee (VSACC) under animal care protocol number AC17-0224.

### Biopsy specimen processing.

All biopsy specimens were removed from ATM tubes and processed in an anaerobic chamber (Bactron3000; Sheldon Manufacturing Inc., Cornelius, OR, USA). To limit environmental contamination, the outer layer of the epidermal skin was removed from the biopsy specimens prior to any additional processing. Using sterile scalpels, biopsy specimens were then sectioned longitudinally into 3 approximately equal sections: 1 biopsy specimen section was for anaerobic culture, 1 was for DNA extraction, and 1 was for long-term storage at −80°C.

DD-associated anaerobic bacteria were cultured and isolated from biopsy specimens to determine which bacterial species were present in DD lesions and for collecting isolates to aid in qPCR development. Biopsy specimens were cultured for up to 7 days at 37°C on fastidious anaerobe agar (FAA; Neogen Corporation, Lansing, MI, USA) in an anaerobic chamber (5% CO_2_, 5% H_2_, and 90% N_2_) to isolate and identify DD-associated species of non-Treponema anaerobic bacteria. FAA medium was supplemented with 1 μg/ml vitamin K_1_ (Sigma-Aldrich, St. Louis, MO, USA), 5 μg/ml hemin (Sigma-Aldrich, St. Louis, MO, USA), and 5% defibrinated sheep blood (Cedarlane Laboratories, Burlington, Ontario, Canada). Biopsy specimens were first smeared to cover one-quarter of the area on each FAA plate and then streaked across the rest of the plate to obtain separate colonies. After anaerobic incubation, colonies with different morphologies were subcultured to isolate single colonies, which were subsequently identified by Sanger sequencing of the full-length 16S rRNA gene using primers 27F (5′-AGAGTTTGATCMTGGCTC-3′) and 1392R (5′-CGGAACATGTGMGGCGGG-3′). Colony PCR assay mixtures before sequencing had a total volume of 25 μl and contained TopTaq master mix (Qiagen, Hilden, Germany) and primers at 400 nM. PCR cycle conditions included an initial denaturation step at 95°C for 5 min, followed by 35 cycles of 95°C for 30 s, 58°C for 30 s, and 72°C for 90 s and a final step at 72°C for 10 min. Sanger sequences were aligned against the BLAST nt database, and bacterial species were identified when the full-length 16S rRNA sequence identity was greater than 98.65%.

Biopsy specimens were weighed before DNA extraction, and up to 25 mg of biopsy tissue was used for extraction. Biopsy specimens were incubated at 56°C overnight in a solution containing 40 μl of proteinase K, 180 μl of tissue lysis (ATL) buffer, and 20 mg/ml of lysozyme (Sigma-Aldrich, St. Louis, MO, USA) until tissue lysis was complete. The lysis mixture was then transferred to a tube containing 200 mg of 0.1-mm zirconia-silica beads for 3 min of uninterrupted bead beating, and DNA extraction was then performed using the Qiagen DNeasy blood and tissue kit (Qiagen, Hilden, Germany) according to the manufacturer’s recommendations for animal tissue. DNA extraction controls were performed without biopsy tissue to identify contaminants during the extraction process. DNA was stored at −80°C in DNase/RNase-free water until use for sequencing and qPCR.

### Deep amplicon sequencing and analysis.

The V3-V4 hypervariable region of the 16S rRNA gene was sequenced from purified biopsy specimen DNA, along with DNA extraction and blank (DNase/RNase-free water) controls. Primers used for sequencing are described in Table S1 in the supplemental material. In a nested reaction to generate amplicons for sequencing, 15 cycles of amplification were performed using primers 8F (AGAGTTTGATCCTGGCTCAG) and 926R (CCGTCAATTCCTTTRAGTTT), followed by 25 cycles with primers 341F and 806R (33) to amplify the V3-V4 hypervariable region. PCR products were visualized on agarose gels, and positive amplicons were sequenced on the Illumina MiSeq platform (v3; 600 cycles; 2 by 300 nucleotides [nt]). Amplification and sequencing of biopsy specimen DNA were performed by the McMaster Genome Facility (Hamilton, ON, Canada).

10.1128/mSystems.00708-21.3 TABLE S1 Primers and probes used to sequence and quantify DD-associated bacteria. Download Table S1, DOCX file, 0.01 MB. Copyright © 2021 Caddey et al. 2021 Caddey et al. https://creativecommons.org/licenses/by/4.0/ This content is distributed under the terms of the Creative Commons Attribution 4.0 International license.

Demultiplexed reads were analyzed and processed using the DADA2 R package v.1.14.1 (34). Forward and reverse reads were truncated approximately after the average Phred score dropped below 30. Shorter reads and reads with ambiguous nucleotides were removed. Next, amplicon sequence variants (ASVs) were inferred, and the paired-end reads were then merged with a minimum overlap of 30 nt. After the removal of chimeric sequences, ASVs were taxonomically classified using the SILVA v.132 database (35). Species classification for Treponema, *Bacteroides*, *Mycoplasma*, *Porphyromonas*, and *Fusobacterium* was performed locally with BLAST+ v.2.10.0 (36) against the NCBI 16S rRNA RefSeq database (37), using the top hit and a 97% identity cutoff against full-length sequences for species classification.

For beta-diversity analysis, samples were first rarefied to minimum sequencing depth (894 sequences) in order to retain as many samples as possible. Weighted and unweighted UniFrac distances were calculated and analyzed using principal-coordinate analysis (PCoA) to identify differences in microbial composition between samples. Variation in microbial composition relative to the M stage was measured by PERMANOVA with 999 permutations and was considered significant with a *P* value of less than 0.05. All diversity analyses were performed using vegan v.2.5.6 (38). Relative abundances were calculated for all samples and then displayed as the mean percent relative abundance for each M stage. DESeq2 v.1.26.0 (39) was used to normalize the sequencing depth and identify DD-associated taxa across M stages, and associations were considered significant at a *P* value of less than 0.01. All analyses of deep amplicon sequencing reads were conducted in R v.3.5.3.

### Whole-genome sequencing and analysis.

Whole-genome sequencing (WGS) was performed for isolates of *Fusobacterium* sp., Porphyromonas levii, and Bacteroides pyogenes (Table S2) to identify species-specific genes for qPCR targets. DNA was extracted from these isolates using the Qiagen DNeasy blood and tissue kit (Qiagen, Hilden, Germany), according to the manufacturer’s instructions, and normalized to 5 ng/μl using the Qubit dsDNA (double-stranded DNA) HS kit (Life Technologies, Carlsbad, CA, USA). Sequencing and draft genome assembly were conducted as described previously by Derakhshani et al. (40) for barcoded Illumina HiSeq reads. Briefly, sequencing libraries were prepared using the NEBNext Ultra II FS DNA library prep kit for Illumina (New England BioLabs [NEB], Ipswich, MA, USA). Libraries were sequenced at the McMaster Genome Facility (Hamilton, ON, Canada) on the Illumina HiSeq 2500 system (2 by 250 nt). *De novo* assembly of draft genomes was performed using Unicycler v.0.4.8 (41). Assembled genomes were then annotated using Prokka v.1.14.5 (42).

Species-specific genes, defined as genes that are present in all known strains of a particular species but absent in other bacteria, were identified for Porphyromonas levii, *Fusobacterium* sp., and Bacteroides pyogenes, according to methods described previously by Naushad et al. (43). Briefly, potentially unique genes were identified by performing a BLASTn search of all open reading frames (ORFs) of a species of interest against an in-house database containing newly sequenced genomes and representative genomes from known bacterial species. The ORFs that were detected in a single species but not identified in any other species were considered unique genes. The specificity and copy number of candidate species-specific genes were confirmed by a BLASTn search against the draft genomes and publicly available genomes of each species.

### Multiplex qPCR development and validation.

A multiplex qPCR targeting species-specific genes of Porphyromonas levii, *Fusobacterium* sp. (undefined species [BioSample accession number SAMN16729900]), and Bacteroides pyogenes was developed and validated for absolute quantification directly from DD biopsy specimen DNA. Species-specific genes for each target species were IX289_000224 (*Fusobacterium* sp.), IX335_000626 (*P. levii*), and IX319_002165 (*B. pyogenes*). Only one copy of each gene is assumed per genome based on BLAST searches of each species-specific gene against all genomes available for each species. Primers and fluorescent probes were designed using Primer3 v.4.1.0 and are shown in Table S1. Primer and probe sequences were designed so that primers had a melting temperature (*T_m_*) of approximately 60°C and probes had a *T_m_* from 67°C to 70°C, amplifying targets of between 75 and 175 bp. The GC content was between 40 and 60% for all oligonucleotides. Primer specificities in singleplex reactions were validated by melt curve analysis, gel electrophoresis, and Sanger sequencing of the qPCR product. The primer annealing temperature was optimized in a temperature gradient, and melt curve analysis ensured specificity. Multiplex reactions with all primer/probe combinations were optimized when singleplex and multiplex results run in parallel were within 1 *C_T_* (threshold cycle) value, the standard deviations (SD) among triplicate *C_T_* values were less than 0.5, and the reaction efficiencies were all between 90 and 110%. DNAs purified from each species (BioSample accession numbers SAMN16729910 for *P. levii*, SAMN16729900 for *Fusobacterium* sp., and SAMN16729906 for *B. pyogenes*) were used as qPCR standards. The multiplex qPCR was further validated for absolute quantification directly from biopsy specimen DNA through a spike-in experiment in order to determine if accurate absolute quantification was possible using this multiplex qPCR. Standard DNA (5 × 10^4^ copies) for each species was spiked into five biopsy specimen DNA samples that did not have any previously detectable species DNA, and the qPCR output (triplicate copy numbers of the target gene) was compared to the expected 5 × 10^4^ gene copies. The final multiplex qPCR mixture contained all primers at 500 μM, probes at 250 μM, 10 μl TaqMan Fast advanced master mix (Applied Biosystems, Foster City, CA, USA), 2 μl of template biopsy specimen DNA (20 ng), and H_2_O for a total reaction volume of 20 μl. Final multiplex qPCR cycling conditions were 50°C for 2 min, 95°C for 20 s, 40 cycles of 95°C for 10 s, and 59.6°C for 30 s.

### Quantitative real-time PCR and analysis.

Absolute quantification by qPCR (CFX96 real-time system; Bio-Rad Laboratories Inc., Hercules, CA, USA) was performed for microbiota strongly associated with DD lesions. In total, 3 different qPCR assays were used in this study: a multiplex qPCR developed by Beninger et al. (22) targeting 4 different Treponema spp. highly prevalent in DD, a qPCR developed by Witcomb et al. (44) targeting Fusobacterium necrophorum, and the multiplex qPCR developed in this study targeting *P. levii*, *Fusobacterium* sp., and *B. pyogenes*. Standards for the Treponema qPCR were prepared as plasmid copy numbers as described previously by Beninger et al. (22), whereas genomic DNAs purified from DD isolates were used for the other qPCR assays (BioSample accession numbers SAMN16729910 for *P. levii*, SAMN16729900 for *Fusobacterium* sp., SAMN16729906 for *B. pyogenes*, and SAMN16729904 for F. necrophorum). All standards were measured with the Qubit dsDNA HS kit (Life Technologies, Carlsbad, CA, USA) before each reaction. Purified biopsy specimen DNA was normalized to 10 ng/μl prior to qPCR. All qPCRs required an efficiency of between 90 and 110%, and all no-template controls were negative. All DNA extraction controls were tested with each qPCR.

All absolute abundances were normalized by the tissue biopsy specimen weight used for DNA extraction so that bacterial quantities were compared as copy numbers per milligram of biopsy tissue. Nonparametric Kruskal-Wallis tests and *post hoc* Mann-Whitney U tests were used to identify significant differences between mean species abundances. Correlation networks were generated to determine the direction and strength of pairwise associations between individual bacterial species in DD lesions. Correlation matrices were generated by calculating pairwise Spearman correlations between natural-log-transformed copy numbers of each bacterial species. Only significant correlations were included in the network analysis. Networks were visualized using the R package igraph v 1.2.5 (45). All *P* values were corrected for multiple hypotheses with the Benjamini-Hochberg method (46), and *P* values of less than 0.05 were considered statistically significant. All analyses were conducted in R v.3.5.3.

### Data availability.

Raw fastq reads generated from deep amplicon sequencing are accessible at the NCBI SRA database (www.ncbi.nlm.nih.gov/sra) under BioProject accession number PRJNA664530. WGS data for all strains sequenced in this study are accessible at the NCBI GenBank database (www.ncbi.nlm.nih.gov/genbank) under BioProject accession number PRJNA676053.

## References

[1] Cheli R, Mortellaro C. 1974. La dermatite digitale del bovino, p 208–213. *In* P. Gallarti (ed), Proceedings of the 8th International Conference on Diseases of Cattle, Piacenza, Milan, Italy.

[2] Orsel K, Plummer P, Shearer J, De Buck J, Carter SD, Guatteo R, Barkema HW. 2018. Missing pieces of the puzzle to effectively control digital dermatitis. Transbound Emerg Dis 65:186–198. doi:10.1111/tbed.12729. 29124910

[3] Read DH, Walker RL. 1998. Papillomatous digital dermatitis (footwarts) in California dairy cattle: clinical and gross pathologic findings. J Vet Diagn Invest 10:67–76. doi:10.1177/104063879801000112. 9526863

[4] Gomez A, Cook NB, Socha MT, Döpfer D. 2015. First-lactation performance in cows affected by digital dermatitis during the rearing period. J Dairy Sci 98:4487–4498. doi:10.3168/jds.2014-9041. 25958279

[5] Dolecheck KA, Overton MW, Mark TB, Bewley JM. 2019. Use of a stochastic simulation model to estimate the cost per case of digital dermatitis, sole ulcer, and white line disease by parity group and incidence timing. J Dairy Sci 102:715–730. doi:10.3168/jds.2018-14901. 30415843

[6] Solano L, Barkema HW, Mason S, Pajor EA, LeBlanc SJ, Orsel K. 2016. Prevalence and distribution of foot lesions in dairy cattle in Alberta, Canada. J Dairy Sci 99:6828–6841. doi:10.3168/jds.2016-10941. 27236761

[7] Yang DA, Johnson WO, Müller KR, Gates MC, Laven RA. 2019. Estimating the herd and cow level prevalence of bovine digital dermatitis on New Zealand dairy farms: a Bayesian superpopulation approach. Prev Vet Med 165:76–84. doi:10.1016/j.prevetmed.2019.02.014. 30851931

[8] Kulow M, Merkatoris P, Anklam KS, Rieman J, Larson C, Branine M, Döpfer D. 2017. Evaluation of the prevalence of digital dermatitis and the effects on performance in beef feedlot cattle under organic trace mineral supplementation. J Anim Sci 95:3435–3444. doi:10.2527/jas2017.1512. 28805925

[9] Krull AC, Shearer JK, Gorden PJ, Scott HM, Plummer PJ. 2016. Digital dermatitis: natural lesion progression and regression in Holstein dairy cattle over 3 years. J Dairy Sci 99:3718–3731. doi:10.3168/jds.2015-10535. 26923049

[10] Döpfer D, Koopmans A, Meijer FA, Szakáll I, Schukken YH, Klee W, Bosma RB, Cornelisse JL, Van Asten AJAM, Ter Huurne AAHM. 1997. Histological and bacteriological evaluation of digital dermatitis in cattle, with special reference to spirochaetes and *Campylobacter faecalis*. Vet Rec 140:620–623. doi:10.1136/vr.140.24.620. 9228692

[11] Brandt S, Apprich V, Hackl V, Tober R, Danzer M, Kainzbauer C, Gabriel C, Stanek C, Kofler J. 2011. Prevalence of bovine papillomavirus and *Treponema* DNA in bovine digital dermatitis lesions. Vet Microbiol 148:161–167. doi:10.1016/j.vetmic.2010.08.031. 20875931

[12] Evans NJ, Brown JM, Demirkan I, Murray RD, Birtles RJ, Hart CA, Carter SD. 2009. *Treponema pedis* sp. nov., a spirochaete isolated from bovine digital dermatitis lesions. Int J Syst Evol Microbiol 59:987–991. doi:10.1099/ijs.0.002287-0. 19406779

[13] Klitgaard K, Bretó AF, Boye M, Jensen TK. 2013. Targeting the treponemal microbiome of digital dermatitis infections by high-resolution phylogenetic analyses and comparison with fluorescent in situ hybridization. J Clin Microbiol 51:2212–2219. doi:10.1128/JCM.00320-13. 23658264 PMC3697659

[14] Klitgaard K, Boye M, Capion N, Jensen TK. 2008. Evidence of multiple *Treponema* phylotypes involved in bovine digital dermatitis as shown by 16S rRNA gene analysis and fluorescence in situ hybridization. J Clin Microbiol 46:3012–3020. doi:10.1128/JCM.00670-08. 18562583 PMC2546760

[15] Nielsen MW, Strube ML, Isbrand A, Al-Medrasi WDHM, Boye M, Jensen TK, Klitgaard K. 2016. Potential bacterial core species associated with digital dermatitis in cattle herds identified by molecular profiling of interdigital skin samples. Vet Microbiol 186:139–149. doi:10.1016/j.vetmic.2016.03.003. 27016768

[16] Capion N, Boye M, Ekstrøm CT, Jensen TK. 2012. Infection dynamics of digital dermatitis in first-lactation Holstein cows in an infected herd. J Dairy Sci 95:6457–6464. doi:10.3168/jds.2012-5335. 22939796

[17] Schlafer S, Nordhoff M, Wyss C, Strub S, Hübner J, Gescher DM, Petrich A, Göbel UB, Moter A. 2008. Involvement of *Guggenheimella bovis* in digital dermatitis lesions of dairy cows. Vet Microbiol 128:118–125. doi:10.1016/j.vetmic.2007.09.024. 18024006

[18] Yano T, Moe KK, Yamazaki K, Ooka T, Hayashi T, Misawa N. 2010. Identification of candidate pathogens of papillomatous digital dermatitis in dairy cattle from quantitative 16S rRNA clonal analysis. Vet Microbiol 143:352–362. doi:10.1016/j.vetmic.2009.12.009. 20036086

[19] Staton GJ, Sullivan LE, Blowey RW, Carter SD, Evans NJ. 2020. Surveying bovine digital dermatitis and non-healing bovine foot lesions for the presence of *Fusobacterium necrophorum*, *Porphyromonas endodontalis* and *Treponema pallidum*. Vet Rec 186:450. doi:10.1136/vr.105628. 32066637 PMC7279135

[20] Krull AC, Shearer JK, Gorden PJ, Cooper VL, Phillips GJ, Plummer PJ. 2014. Deep sequencing analysis reveals temporal microbiota changes associated with development of bovine digital dermatitis. Infect Immun 82:3359–3373. doi:10.1128/IAI.02077-14. 24866801 PMC4136199

[21] Johnson JS, Spakowicz DJ, Hong BY, Petersen LM, Demkowicz P, Chen L, Leopold SR, Hanson BM, Agresta HO, Gerstein M, Sodergren E, Weinstock GM. 2019. Evaluation of 16S rRNA gene sequencing for species and strain-level microbiome analysis. Nat Commun 10:5029. doi:10.1038/s41467-019-13036-1. 31695033 PMC6834636

[22] Beninger C, Naqvi SA, Naushad S, Orsel K, Luby C, Derakhshani H, Khafipour E, De Buck J. 2018. Associations between digital dermatitis lesion grades and the quantities of four *Treponema* species. Vet Res 49:111. doi:10.1186/s13567-018-0605-z. 30373670 PMC6206660

[23] Sullivan LE, Carter SD, Blowey R, Duncan JS, Grove-White D, Evans NJ. 2013. Digital dermatitis in beef cattle. Vet Rec 173:582. doi:10.1136/vr.101802. 24106250

[24] Sullivan LE, Evans NJ, Blowey RW, Grove-White DH, Clegg SR, Duncan JS, Carter SD. 2015. A molecular epidemiology of treponemes in beef cattle digital dermatitis lesions and comparative analyses with sheep contagious ovine digital dermatitis and dairy cattle digital dermatitis lesions. Vet Microbiol 178:77–87. doi:10.1016/j.vetmic.2015.04.011. 25937315

[25] Moreira TF, Facury Filho EJ, Carvalho AU, Strube ML, Nielsen MW, Klitgaard K, Jensen TK. 2018. Pathology and bacteria related to digital dermatitis in dairy cattle in all year round grazing system in Brazil. PLoS One 13:e0193870. doi:10.1371/journal.pone.0193870. 29513739 PMC5841792

[26] Zinicola M, Lima F, Lima S, Machado V, Gomez M, Döpfer D, Guard C, Bicalho R. 2015. Altered microbiomes in bovine digital dermatitis lesions, and the gut as a pathogen reservoir. PLoS One 10:e0120504. doi:10.1371/journal.pone.0120504. 25781328 PMC4362943

[27] Marcatili P, Nielsen MW, Sicheritz-Pontén T, Jensen TK, Schafer-Nielsen C, Boye M, Nielsen M, Klitgaard K. 2016. A novel approach to probe host-pathogen interactions of bovine digital dermatitis, a model of a complex polymicrobial infection. BMC Genomics 17:987. doi:10.1186/s12864-016-3341-7. 27908274 PMC5142292

[28] Van Metre DC. 2017. Pathogenesis and treatment of bovine foot rot. Vet Clin North Am Food Anim Pract 33:183–194. doi:10.1016/j.cvfa.2017.02.003. 28579042

[29] Lockhart JS, Buret AG, Ceri H, Storey DG, Anderson SJ, Morck DW. 2017. Mixed species biofilms of *Fusobacterium necrophorum* and *Porphyromonas levii* impair the oxidative response of bovine neutrophils in vitro. Anaerobe 47:157–164. doi:10.1016/j.anaerobe.2017.05.008. 28526497

[30] Brooks JP, Edwards DJ, Harwich MD, Jr, Rivera MC, Fettweis JM, Serrano MG, Reris RA, Sheth NU, Huang B, Girerd P, Vaginal Microbiome Consortium, Strauss JF, III, Jefferson KK, Buck GA. 2015. The truth about metagenomics: quantifying and counteracting bias in 16S rRNA studies. BMC Microbiol 15:66. doi:10.1186/s12866-015-0351-6. 25880246 PMC4433096

[31] Jian C, Luukkonen P, Yki-Järvinen H, Salonen A, Korpela K. 2020. Quantitative PCR provides a simple and accessible method for quantitative microbiota profiling. PLoS One 15:e0227285. doi:10.1371/journal.pone.0227285. 31940382 PMC6961887

[32] Berry SL, Read DH, Famula TR, Mongini A, Döpfer D. 2012. Long-term observations on the dynamics of bovine digital dermatitis lesions on a California dairy after topical treatment with lincomycin HCl. Vet J 193:654–658. doi:10.1016/j.tvjl.2012.06.048. 22892182

[33] Klindworth A, Pruesse E, Schweer T, Peplies J, Quast C, Horn M, Glöckner FO. 2013. Evaluation of general 16S ribosomal RNA gene PCR primers for classical and next-generation sequencing-based diversity studies. Nucleic Acids Res 41:e1. doi:10.1093/nar/gks808. 22933715 PMC3592464

[34] Callahan BJ, McMurdie PJ, Rosen MJ, Han AW, Johnson AJA, Holmes SP. 2016. DADA2: high-resolution sample inference from Illumina amplicon data. Nat Methods 13:581–583. doi:10.1038/nmeth.3869. 27214047 PMC4927377

[35] Quast C, Pruesse E, Yilmaz P, Gerken J, Schweer T, Yarza P, Peplies J, Glöckner FO. 2013. The SILVA ribosomal RNA gene database project: improved data processing and Web-based tools. Nucleic Acids Res 41:D590–D596. doi:10.1093/nar/gks1219. 23193283 PMC3531112

[36] Camacho C, Coulouris G, Avagyan V, Ma N, Papadopoulos J, Bealer K, Madden TL. 2009. BLAST+: architecture and applications. BMC Bioinformatics 10:421. doi:10.1186/1471-2105-10-421. 20003500 PMC2803857

[37] O’Leary NA, Wright MW, Brister JR, Ciufo S, Haddad D, McVeigh R, Rajput B, Robbertse B, Smith-White B, Ako-Adjei D, Astashyn A, Badretdin A, Bao Y, Blinkova O, Brover V, Chetvernin V, Choi J, Cox E, Ermolaeva O, Farrell CM, Goldfarb T, Gupta T, Haft D, Hatcher E, Hlavina W, Joardar VS, Kodali VK, Li W, Maglott D, Masterson P, McGarvey KM, Murphy MR, O’Neill K, Pujar S, Rangwala SH, Rausch D, Riddick LD, Schoch C, Shkeda A, Storz SS, Sun H, Thibaud-Nissen F, Tolstoy I, Tully RE, Vatsan AR, Wallin C, Webb D, Wu W, Landrum MJ, Kimchi A, et al. 2016. Reference Sequence (RefSeq) database at NCBI: current status, taxonomic expansion, and functional annotation. Nucleic Acids Res 44:D733–D745. doi:10.1093/nar/gkv1189. 26553804 PMC4702849

[38] Dixon P. 2003. VEGAN, a package of R functions for community ecology. J Veg Sci 14:927–930. doi:10.1111/j.1654-1103.2003.tb02228.x.

[39] Love MI, Huber W, Anders S. 2014. Moderated estimation of fold change and dispersion for RNA-seq data with DESeq2. Genome Biol 15:550. doi:10.1186/s13059-014-0550-8. 25516281 PMC4302049

[40] Derakhshani H, Bernier SP, Marko VA, Surette MG. 2020. Completion of draft bacterial genomes by long-read sequencing of synthetic genomic pools. BMC Genomics 21:519. doi:10.1186/s12864-020-06910-6. 32727443 PMC7392658

[41] Wick RR, Judd LM, Gorrie CL, Holt KE. 2017. Unicycler: resolving bacterial genome assemblies from short and long sequencing reads. PLoS Comput Biol 13:e1005595. doi:10.1371/journal.pcbi.1005595. 28594827 PMC5481147

[42] Seemann T. 2014. Prokka: rapid prokaryotic genome annotation. Bioinformatics 30:2068–2069. doi:10.1093/bioinformatics/btu153. 24642063

[43] Naushad HS, Lee B, Gupta RS. 2014. Conserved signature indels and signature proteins as novel tools for understanding microbial phylogeny and systematics: identification of molecular signatures that are specific for the phytopathogenic genera *Dickeya*, *Pectobacterium* and *Brenneria*. Int J Syst Evol Microbiol 64:366–383. doi:10.1099/ijs.0.054213-0. 24505075

[44] Witcomb LA, Green LE, Kaler J, Ul-Hassan A, Calvo-Bado LA, Medley GF, Grogono-Thomas R, Wellington EMH. 2014. A longitudinal study of the role of *Dichelobacter nodosus* and *Fusobacterium necrophorum* load in initiation and severity of footrot in sheep. Prev Vet Med 115:48–55. doi:10.1016/j.prevetmed.2014.03.004. 24703249 PMC4029074

[45] Csardi G, Nepusz T. 2006. The igraph software package for complex network research. Interjournal Complex Syst 2006:1695.

[46] Benjamini Y, Hochberg Y. 1995. Controlling the false discovery rate: a practical and powerful approach to multiple testing. J R Stat Soc Series B Stat Methodol 57:289–300.

