# Supplementary figures and images for: Identification and Quantification of Bovine Digital Dermatitis-Associated Microbiota across Lesion Stages in Feedlot Beef Cattle
Source: mSystems. 2021 Jul 27;6(4):e00708-21. doi: 10.1128/mSystems.00708-21 (PMC8409723; doi:10.1128/mSystems.00708-21)

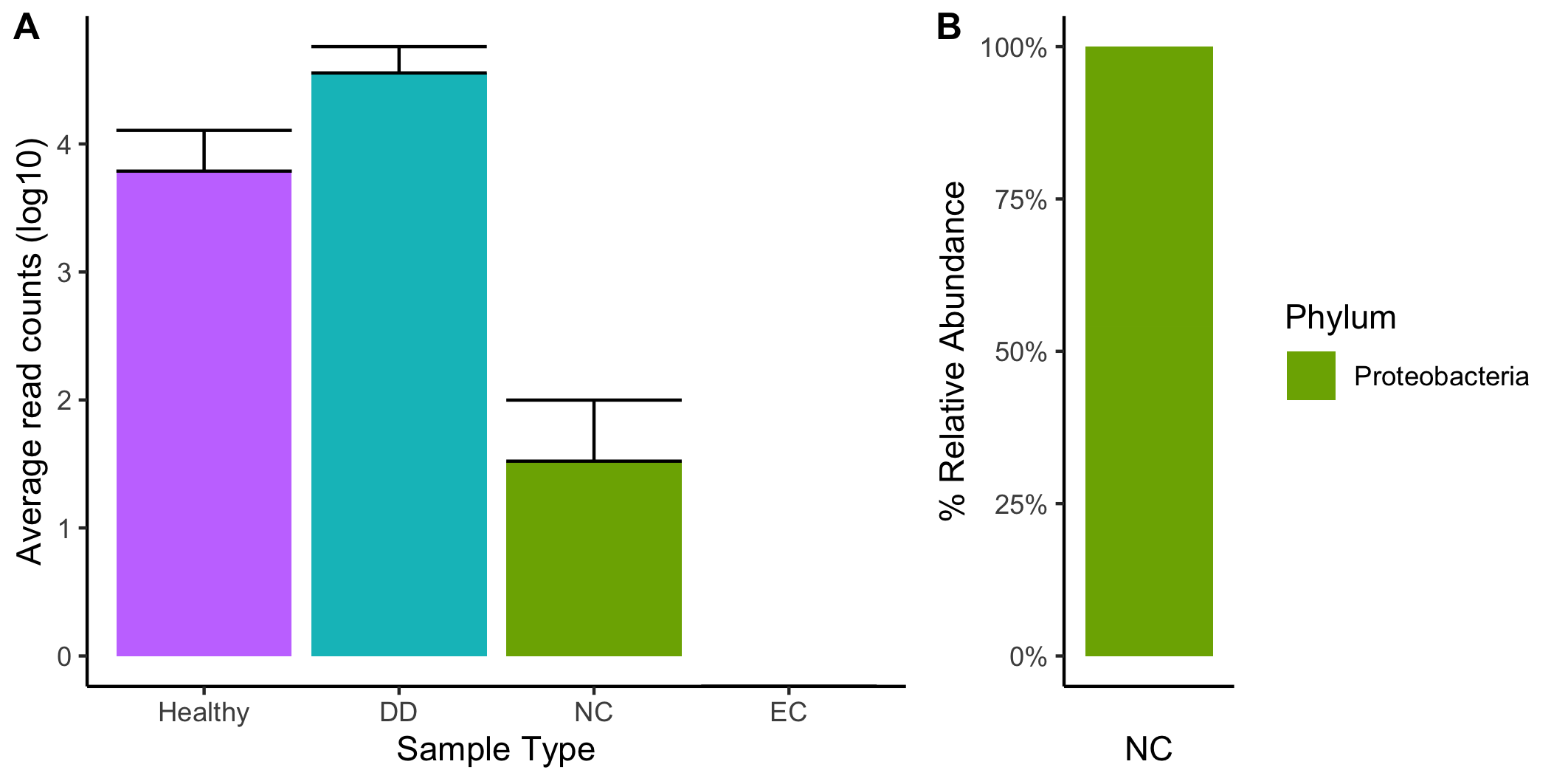

Supplement: FIG S1 [file msystems.00708-21-sf001.tif]

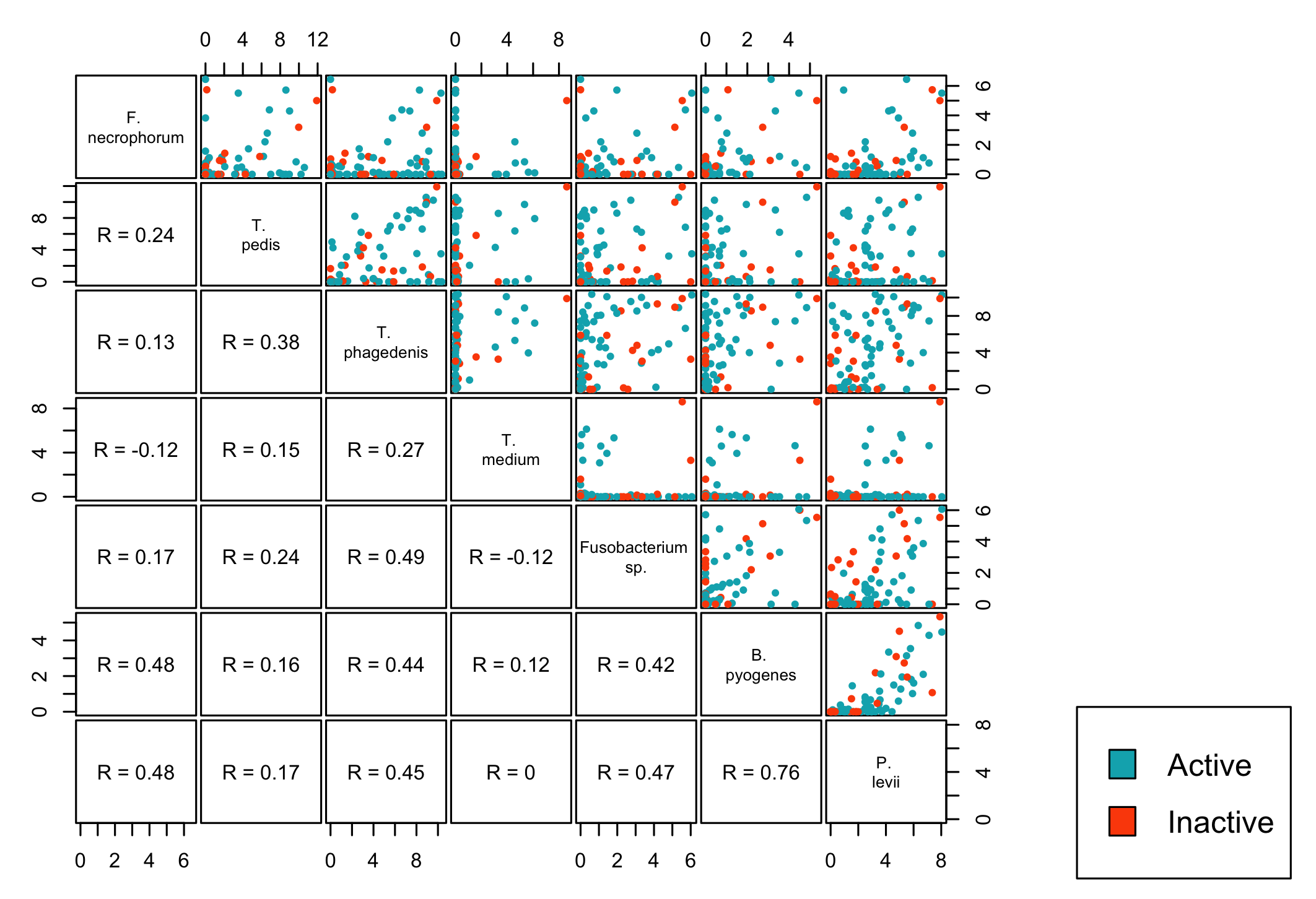

Supplement: FIG S2 [file msystems.00708-21-sf002.tif]
