# Supplementary material for: Identification and Quantification of Bovine Digital Dermatitis-Associated Microbiota across Lesion Stages in Feedlot Beef Cattle
Source: mSystems. 2021 Jul 27;6(4):e00708-21. doi: 10.1128/mSystems.00708-21 (PMC8409723; doi:10.1128/mSystems.00708-21)
Supplement: TABLE S2 [file msystems.00708-21-st002.docx]

| Lesion stage | *B. pyogenes* | Fusobacterium sp. | *F. necrophorum* | *P. levii* | *P. somerae* | *Porphyromonas* sp. | *Prevotella* sp. |
| --- | --- | --- | --- | --- | --- | --- | --- |
| M0 | 0 | 0 | 0 | 0 | 0 | 0 | 0 |
| M1 | 7 | 3 | 1 | 1 | 1 | 4 | 0 |
| M2 | 4 | 13 | 1 | 13 | 1 | 10 | 1 |
| M3 | 1 | 1 | 1 | 1 | 0 | 0 | 0 |
| M4 | 2 | 2 | 1 | 4 | 0 | 2 | 0 |
| M4.1 | 3 | 4 | 0 | 3 | 3 | 5 | 0 |
