# Supplementary material for: Identification and Quantification of Bovine Digital Dermatitis-Associated Microbiota across Lesion Stages in Feedlot Beef Cattle
Source: mSystems. 2021 Jul 27;6(4):e00708-21. doi: 10.1128/mSystems.00708-21 (PMC8409723; doi:10.1128/mSystems.00708-21)
Supplement: TABLE S3 [file msystems.00708-21-st003.docx]

|  | *B. pyogenes* | *Fusobacterium* sp*.* | *P. levii* |
| --- | --- | --- | --- |
| Target DNA copies spiked-in | 5.0 x 10^4^ | 5.0 x 10^4^ | 5.0 x 10^4^ |
| Target DNA copies in biopsy | 0 ± 0 | 0 ± 0 | 0 ± 0 |
| Mean ± SD target DNA copies in biopsy after spike-in | 5.1 x 10^4^ ± 6.9 x 10^3^ | 5.7 x 10^4^ ± 1.1 x 10^4^ | 4.7 x 10^4^ ± 5.8 x 10^3^ |
| Efficiency | 95.7% | 91.3% | 92.5% |
| R-squared | 0.998 | 0.996 | 0.996 |

Efficiency and R-squared values are based on qPCR standard curve for each species reaction
