# Supplementary material for: Identification and Quantification of Bovine Digital Dermatitis-Associated Microbiota across Lesion Stages in Feedlot Beef Cattle
Source: mSystems. 2021 Jul 27;6(4):e00708-21. doi: 10.1128/mSystems.00708-21 (PMC8409723; doi:10.1128/mSystems.00708-21)
Supplement: TABLE S4 [file msystems.00708-21-st004.docx]

| Farm ID (M0) | Tphg | Tped | Tmed | Fn | Fs | Pl | Bp |
| --- | --- | --- | --- | --- | --- | --- | --- |
| H  (n=14) | 0.432 ± 0.690^a^ | 0.570 ± 1.06^a^ | 0.177 ± 0.347^a^ | 0.014 ± 0.041^a^ | 0 ± 0^a^ | 0.112 ± 0.267^a^ | 0.002 ± 0.009^a^ |
| K  (n=14) | 5.96 ± 21.2^b^ | 0.327 ± 0.895^b^ | 8.021 ± 29.0^a^ | 0.053 ± 0.182^a^ | 0 ± 0^a^ | 0.386 ± 0.926^a^ | 0.012 ± 0.044^a^ |
| M^*^  (n=12) | 0.133 ± 0.277^c^ | 0.066 ± 0.112^b^ | 0 ± 0^b^ | 0 ± 0^b^ | 0.015 ± 0.052^a^ | 0.022 ± 0.052^b^ | 0 ± 0^b^ |

Tphg, *T. phagedenis*; Tped, *T. pedis*; Tmed, *T. medium*; Fn, *F. necrophorum*; Fs, *Fusobacterium* sp.; Pl, *P. levii*; Bp, *B. pyogenes*. Different letters within a column indicate significant difference (p < 0.05). * Farm had no reported active cases of DD during sampling. Farms H and K had active cases of DD, with an unknown prevalence, during sampling period.
