# Supplementary material for: Identification and Quantification of Bovine Digital Dermatitis-Associated Microbiota across Lesion Stages in Feedlot Beef Cattle
Source: mSystems. 2021 Jul 27;6(4):e00708-21. doi: 10.1128/mSystems.00708-21 (PMC8409723; doi:10.1128/mSystems.00708-21)
Supplement: TABLE S5 [file msystems.00708-21-st005.docx]

|  | Percent (%) of samples with detectable species present | | | | | | |
| --- | --- | --- | --- | --- | --- | --- | --- |
| Farm ID  (M0) | Tphg | Tped | Tmed | Fn | Fs | Pl | Bp |
| H  (n=14) | 71 | 43 | 43 | 14 | 0 | 43 | 7 |
| K  (n=14) | 71 | 21 | 29 | 14 | 0 | 50 | 7 |
| M^*^  (n=12) | 58 | 33 | 0 | 0 | 8 | 17 | 0 |
| Total (n=40) | 68 | 32 | 25 | 10 | 2 | 38 | 5 |

^a^ Detectable by species-specific qPCR. ^*^ Farm had no reported active cases of DD during sampling. Farms H and K had active cases of DD during sampling period, but with an unknown prevalence.
