# Supplementary material for: Identification and Quantification of Bovine Digital Dermatitis-Associated Microbiota across Lesion Stages in Feedlot Beef Cattle
Source: mSystems. 2021 Jul 27;6(4):e00708-21. doi: 10.1128/mSystems.00708-21 (PMC8409723; doi:10.1128/mSystems.00708-21)
Supplement: TABLE S1 [file msystems.00708-21-st001.docx]

| Primer | Sequence (5’–3’) | Target | Reference |
| --- | --- | --- | --- |
| 341F | CCTACGGGNGGCWGCAG | Bacteria | Klindworth et al. 2013 (26) |
| 806R | GGACTACHVGGGTWTCTAAT | Bacteria | Klindworth et al*.* 2013 (26) |
| FusoF | TCTTTCAATGCTGGGATGCTCT | *Fusobacterium* sp. | This study |
| FusoR | TGATGGTCCACAATTCTCTCTACA | *Fusobacterium* sp. | This study |
| PlevF | GGGTGTAGTGCCTACAATAG | *Porphyromonas levii* | This study |
| PlevR | CCTGAGAAGAGCAGATAGTG | *Porphyromonas levii* | This study |
| BpyoF | ATTGGCGCTTGTCTCCTACC | *Bacteroides pyogenes* | This study |
| BpyoR | TATTCATCCATCGTGCGGCC | *Bacteroides pyogenes* | This study |
| FnecF | ﻿AACCTCCGGCAGAAGAAAAATT | *Fusobacterium necrophorum* | Witcomb et al*.* 2014 (37) |
| FnecR | ﻿CGTGAGGCATACGTAGAGAACTGT | *Fusobacterium necrophorum* | Witcomb et al*.* 2014 (37) |
| TmedF | AAAGCGCTACGAATCCTAAG | *Treponema medium* | Beninger et al*.* 2018 (22) |
| TmedR | ATCATTACCCGTCCACAAAG | *Treponema medium* | Beninger et al*.* 2018 (22) |
| TphgF | CCCGCAGGAAGGTATAATC | *Treponema phagedenis* | Beninger et al*.* 2018 (22) |
| TphgR | CACAGCTGTTGTGGTATTAAG | *Treponema phagedenis* | Beninger et al*.* 2018 (22) |
| TpedF | ACACCGATTGTACTGAATGA | *Treponema pedis* | Beninger et al*.* 2018 (22) |
| TpedR | CCACGAGCTTTCTACAGATT | *Treponema pedis* | Beninger et al*.* 2018 (22) |
| TdentF | GGAAACTTAGGAATTCGATATGTAG | *Treponema denticola* | Beninger et al*.* 2018 (22) |
| TdentR | CCTTCTTTAGTTTCTTTGTGAGG | *Treponema denticola* | Beninger et al*.* 2018 (22) |
| Probe | Sequence (5’–3’) | Target | Reference |
| FusoP | HEX™ / CTCACTTTTGCACTTATTTCCTGCACTGA / 3' IB^®^FQ | *Fusobacterium* sp. | This study |
| PlevP | TxR^®^-X NHS / CTTGTCACCATCAAAGGCGGCG / 3' IB^®^FQ | *Porphyromonas levii* | This study |
| BpyoP | 6-FAM™ / CTGACAGACGAAACCCTCAGCAGAATACT / IB^®^FQ | *Bacteroides pyogenes* | This study |
| FnecP | ﻿ 6-FAM™ / TCGAACATCTCTCGCTTTTTCCCCGA / BHQ-1 | *Fusobacterium necrophorum* | Witcomb et al*.* 2014 (37) |
| TmedP | CAL Fluor^®^ Red 610 / TGCACCCTTGTTTACTACTGCACAGCC / BHQ-2 | *Treponema medium* | Beninger et al*.* 2018 (22) |
| TphgP | HEX™ / AATCCGCCTACGACTGCGATACCA / IB^®^FQ | *Treponema phagedenis* | Beninger et al*.* 2018 (22) |
| TpedP | 6-FAM™ / ACTACACGTGGAGTACCGAATGCT / IB^®^FQ | *Treponema pedis* | Beninger et al*.* 2018 (22) |
| TdentP | Quasar^®^ 670 / AGCATACAGCGATTATAACAAAGCCCTCGA / BHQ-2 | *Treponema denticola* | Beninger et al*.* 2018 (22) |
